# Development and validation of conventional and TaqMan real-time PCR for the detection of *Trichoderma afroharzianum* causing corn ear rot

**DOI:** 10.1038/s41598-026-51199-2

**Published:** 2026-05-06

**Authors:** Clovis Douanla-Meli, Annette Pfordt, Andreas von Tiedemann, Gritta Schrader, Bernhard Carl Schäfer

**Affiliations:** 1 Julius Kühn-Institut (JKI) - Federal Research Centre for Cultivated Plants, Institute for National and International Plant Health, Braunschweig, Germany; 2 Plant Pathology and Crop Protection, Georg-August University of Göttingen, Göttingen, Germany

**Keywords:** Pathogenic *Trichoderma*, Taqman PCR, Corn disease, *Trichoderma afroharzianum*, Emerging diseases, Biological techniques, Biotechnology, Microbiology, Molecular biology, Plant sciences

## Abstract

The emergence of a new corn ear rot was recently described in Germany and has since received increased attention following detections in several European and Asian countries. All reports consistently identify *Trichoderma afroharzianum* as the causal agent. The potential impact on maize production, combined with the risk of misdiagnosis as other ear rot diseases, highlights the need for a rapid and accurate diagnostic method for the reliable detection of *T. afroharzianum*. In this study, we developed a PCR-based diagnostic tool including an endpoint PCR and a real-time TaqMan assay targeting the TEF1α and RPB2 loci, respectively. Both assays were validated using a broad panel of *Trichoderma* isolates and other fungal species obtained from maize plants, additional host species from diverse geographic origins, and soils collected from German maize fields. All performance criteria required to demonstrate assay reliability were met. The two PCR assays demonstrated higher analytical specificity and inclusivity, albeit were unable to distinguish between pathogenic and non-pathogenic *T. afroharzianum* isolates. They showed high accuracy and sensitivity, with limits of detection of 1 pg µL⁻¹ for the endpoint PCR and 1 fg µL⁻¹ for the real-time PCR. The real-time assay exhibited amplification efficiencies of 105,5% (R² = 0.977) for pure cultures and 116% (R² = 0.997) for infected maize kernels. Furthermore, both assays reliably detected *T. afroharzianum* in asymptomatic maize cobs following artificial inoculation with pathogenic strains. These methods therefore enable early and reliable detection of *T. afroharzianum* ear rot and allow its discrimination from other maize ear rot diseases. This capability is essential for the development of targeted management strategies to address this emerging threat to maize production.

**Supplementary Information:**

The online version contains supplementary material available at 10.1038/s41598-026-51199-2.

## Introduction

Maize (*Zea mays*) is one of the world’s most important crops and the second most widely produced cereal, serving as an essential food source for both humans and livestock^1,2^. Beyond direct consumption, maize is a highly versatile commodity used in the production of numerous derivatives, including food products, animal feed, biofuels, and a broad range of industrial goods^3^. However, maize production is increasingly challenged by abiotic and biotic stressors that threaten yields and, more broadly, global food security^4^. Abiotic factors such as drought and extreme temperatures are largely driven by climate change^5^, biotic stressors comprise living organisms that negatively affect maize crops and can cause substantial yield losses^6^. These include a wide range of pests and pathogens—bacteria, fungi, viruses, nematodes, insects as well as weeds. Among the most prevalent maize diseases are ear rots, primarily caused by *Fusarium* species, but also by other fungal pathogens such as *Aspergillus*, *Penicillium*, *Nigrospora*, and *Stenocarpella*^7,8^. Previous studies have reported grain yield losses of 10–30% due to ear rot^9,10^, a figure that is likely to rise with the emergence of new ear rot types.

In 2018, a previously unreported Trichoderma ear rot of maize was identified in southern Germany, and *T. afroharzianum* was determined as the causal agent^11^. Initial symptoms consist of white mycelium, which subsequently develops into green to grey-green growth spreading between or over the kernels. A subsequent survey revealed an extensive distribution of the disease throughout Germany^12^. Since then, Trichoderma ear rot has been reported in France, Italy, Austria, and China^12–15^. In all cases, *T. afroharzianum* was identified as the causative agent, supporting the view that this species represents a major pathogen associated with ear rot symptoms under field conditions. In addition to *T. afroharzianum*, pathogenic strains of *T. asperellum* and *T. atroviride* have been reported in India to cause ear rot in maize^16^. Furthermore, *T. afroharzianum* and *T. harzianum* have been associated with post-flowering stalk rot in maize^16^.

Taken together, these findings suggest that an increasing number of *Trichoderma* species may become associated with ear rot and other maize diseases in the future. At the same time, the emergence of *Trichoderma*-associated diseases in maize may have been underestimated due to limited symptom-based differentiation from other ear rot diseases and the insufficient emphasis on diagnostic approaches. This interpretation is supported by the growing number of reports published within a relatively short time frame^11,13,14^. With specific regard to *T. afroharzianum*, this species is globally distributed and commonly occurs in soil, dead wood, and living plants as an endophyte. In addition, it is used as an active ingredient in several commercial biological control products. Its pathogenic lifestyle therefore represents a newly recognised trait of this species and underscores the need for accurate identification. This is particularly important because non-pathogenic species or strains are widely applied in biological control, and their use must be demonstrably safe.

In general, early and efficient diagnosis is a critical prerequisite for the effective management of plant diseases. Accordingly, the rapid, accurate, and reliable detection and identification of *T. afroharzianum* is essential. Morphological characterisation alone is insufficient for the reliable diagnosis of *Trichoderma* species, as many taxa exhibit overlapping phenotypic traits. Consequently, the combination of multilocus phylogenetic analysis (MLPA) and DNA barcoding has become the most effective approach for species-level identification within the genus *Trichoderma*^17–19^. Phylogenetically, *T. afroharzianum* belongs to the Harzianum clade and can be distinguished from closely related species using MLPA. However, MLPA has inherent limitations, most notably the time required to complete the analysis, which restricts its applicability for routine diagnostic purposes^20^. In addition, the generation of pure cultures for DNA sequencing through single-spore isolation can be particularly time-consuming, especially when multiple *Trichoderma* species co-occur on infected maize cobs^12^.

Given the global importance of maize, substantial efforts have been devoted to the development of a wide range of diagnostic technologies, from laboratory-based PCR assays to field-based remote sensing approaches for disease detection^21–23^. Direct methods employing non-invasive imaging sensors have been used predominantly for the diagnosis and monitoring of fungal diseases affecting maize leaves^24–26^. In contrast, applications addressing maize ear rot have so far been largely limited to the detection, monitoring, and quantification of mycotoxin production^27^. The implementation of molecular tools for species- and strain-specific identification, including immunological approaches, fingerprinting techniques, exogenous markers, and PCR-based methods^28,29^, can facilitate the effective detection and differentiation of Trichoderma ear rot. Among these approaches, species-specific PCR has proven to be a particularly effective tool, providing faster, more specific, and more sensitive detection and identification of pathogenic fungi^30,31^.

The primary objective of this study was to develop a specific and sensitive molecular tool to facilitate the detection and identification of *T. afroharzianum*. Primers and hydrolysis probes were designed targeting the TEF1α and RPB2 genes and evaluated for their ability to specifically amplify *T. afroharzianum* from pure cultures as well as from naturally and artificially infected maize kernels. Two assays, a conventional PCR (cPCR) and a real-time TaqMan-based PCR (qPCR), were developed and validated in accordance with the EPPO standard PM 7/98^32^.

## Methods

### Isolates collection and identification

The isolates of *Trichoderma* spp. and other fungi included in this study were primarily derived from maize samples and soil collected from maize fields. Samples of maize cobs and stalks were obtained between 2018 and 2024 from both symptomatic and asymptomatic plants. *Trichoderma* isolates were also recovered from soil originating from fields with and without confirmed Trichoderma ear rot infection. Sampling of soil and maize plant material was conducted throughout Germany in collaboration with farmers willing to contribute to the survey of outbreaks of Trichoderma ear rot disease. Therefore, tacit permission to collect maize was obtained. Samples stored in plastic bags were sent to the laboratory for analysis. The procedures for isolating fungi from maize and soil samples and assigning them to *Trichoderma* followed the methods described by^12^. Additional *Trichoderma* strains were obtained from fungal culture collections, research institutions, and commercial biocontrol products containing *Trichoderma* as active organisms. Isolation of *Trichoderma* spp. from biocontrol products was carried out in accordance with the methodology outlined in^12^. The molecular identification of the new *Trichoderma* isolates used in the present study was conducted in a preceding study¹². This identification was based on sequences from the TEF1α and RPB2 regions (Table S1) by applying the protocol proposed by^19^. All isolates were previously subjected to pathogenicity testing on maize under controlled greenhouse conditions. Details pertaining to the taxonomic identification and pathogenicity are available in^12^. A complete list of fungal strains and their origins used in this study is provided in the Tables 1 and S2.

**Table 1:** Fungal isolates used in this study for testing the inclusivity of the conventional and real-time PCR assays.

Species	Isolate	Year	Source/Host	Location	Assay (target)	
					cPCR	qPCR
*T. afroharzianum* ^P^	AP18TRI1	2018	Symptomatic corncobs	France	+	21.70 ± 0.54
*T. afroharzianum* ^P^	AP18TRI2	2018	Symptomatic corncobs	Germany	+	20.45 ± 0.78
*T. afroharzianum* ^P^	AP18TRI3	2018	Symptomatic corncobs	Germany	+	20.26 ± 0.24
*T. afroharzianum* ^P^	AP19TRI5	2019	Symptomatic corncobs	Germany	+	22.60 ± 0.57
*T. afroharzianum* ^NP^	CBS 124,620	–	*Theobroma cacao*	Peru	+	21.58 ± 0.59
*T. afroharzianum* ^P^	AP20TRI15	2020	Symptomatic corncobs	Germany	+	19.25 ± 0.89
*T. afroharzianum* ^P^	AP20TRI16	2020	Symptomatic corncobs	Germany	+	20.14 ± 0.35
*T. afroharzianum* ^NP^	KG10	-	*Pleurotus ostreatus* substrate	-	+	20.97 ± 0.71
*T. afroharzianum* ^NP^	KG13	-	*P. ostreatus* substrate	-	+	25.83 ± 0.31
*T. afroharzianum* ^NP^	MRI349	-	Biostimulant	-	+	21.53 ± 0.28
*T. afroharzianum* ^NP^	T42	-	*Marchantia polymorpha*	-	+	21.87 ± 0.41
*T. afroharzianum* ^NP^	T138	-	*M. polymorpha*	-	+	21.23 ± 0.62
*T. afroharzianum* ^P^	DISAFATS-1	-	Symptomatic corncobs	Italy	+	19.93 ± 1.08
*T. afroharzianum* ^P^	AP22TRI81	2022	Symptomatic corncobs	Germany	+	21.29 ± 0.57
*T. afroharzianum* ^P^	AP22TRI19	2022	Symptomatic corncobs	Germany	+	22.87 ± 0.51
*T. afroharzianum* ^P^	AP22TRI99	2022	Soil maize field	Germany	+	22.87 ± 0.51
*T. afroharzianum* ^P^	AP22TRI108	2022	Soil maize field	Germany	+	20.82 ± 1.07
*T. afroharzianum* ^P^	AP22TRI118	2022	Soil maize field	Germany	+	19.73 ± 0.82
*T. afroharzianum* ^P^	AP22TRI121	2022	Soil maize field	Germany	+	20.66 ± 0.76
*T. afroharzianum* ^P^	AP22TRI129	2022	Soil maize field	Germany	+	21.11 ± 1.47
*T. afroharzianum* ^P^	AP22TRI130	2022	Soil maize field	Germany	+	19.70 ± 0 .71
*T. afroharzianum* ^P^	AP22TRI131	2022	Soil maize field	Germany	+	23.27 ± 1.76
*T. afroharzianum* ^P^	AP22TRI134	2022	Soil maize field	Germany	+	24.94 ± 0.77
*T. afroharzianum* ^P^	AP23TRI136	2022	Soil maize field	Germany	+	23.36 ± 0.67
*T. afroharzianum* ^P^	AP23TRI221	2023	Soil maize field	Germany	+	24.17 ± 1.75
*T. afroharzianum* ^P^	AP22TRI225	2023	Soil maize field	Germany	+	23.97 ± 1.56
*T. afroharzianum* ^P^	AP24TRI401	2024	Symptomatic corncobs	Germany	+	24.32 ± 1.14
*T. afroharzianum* ^P^	AP24TRI402	2024	Symptomatic corncobs	Germany	+	23.64 ± 1.24
*T. afroharzianum* ^P^	AP24TRI403	2024	Symptomatic corncobs	Germany	+	23.37 ± 1.05
*T. afroharzianum* ^P^	AP24TRI404	2024	Symptomatic corncobs	Germany	+	24.24 ± 0.70
*T. afroharzianum* ^P^	AP14TRI409	2024	Symptomatic corncobs	Germany	+	24.08 ± 0.76
*T. afroharzianum* ^P^	AP14TRI414	2024	Symptomatic corncobs	Germany	+	23.84 ± 1.10
*T. afroharzianum* ^P^	AP14TRI426	2024	Soil maize field	Germany	+	24.19 ± 0.85
*T. afroharzianum* ^P^	AP14TRI430	2024	Soil maize field	Germany	+	22.67 ± 0.08
*T. afroharzianum* ^P^	AP14TRI450	2024	Corncobs	Germany	+	23.34 ± 0.16
*T. afroharzianum* ^P^	AP14TRI458	2024	Corncobs	Germany	+	23.58 ± 1.34
*T. afroharzianum* ^P^	AP14TRI460	2024	Corncobs	Germany	+	24.48 ± 1.23
*T. afroharzianum* ^P^	AP14TRI461	2024	Corncobs	Germany	+	21.70 ± 0.54
*T. afroharzianum* ^P^	AP14TRI462	2024	Corncobs	Germany	+	20.45 ± 0.78

For each isolate following details are provided: taxonomy, name, year of collection, source or host, location and the results of testing with endpoint and real-time PCR. The pathogenicity of all isolates on maize was tested in previous work^12^ in a greenhouse (see legend below).

^P^: Pathogenic; ^NP^: Non-pathogenic on maize as tested in the greenhouse.

In this study, 7 *Trichoderma afroharzianum* isolates (namely, CBS 124620, KG10, KG13, MRI349, T42, T138 and DISAFATS-1) were obtained from a variety of sources, including culture collection, research institute and Biostimulant. The remaining 32 isolates beginning with ‘AP’ were recently generated by Annette Pfordt and subsequently identified by Clovis Douanla-Meli. These samples are deposited under the same nomenclature in the fungal collection of the Department of Crop Sciences, Division of Plant Diseases and Crop Protection at Georg August University of Göttingen in Germany.

### Design of primers and hydrolysis probes

Sequences generated from the TEF1α and RPB2 genes for *Trichoderma* species in the Brevicompactum, Harzianum, Virens and Viride clades including referenced sequences retrieved from the NCBI GenBank database were composed. Alignment was performed in MAFFT v. 7 online version^33^ using the iterative refinement option G-INS-i and manual optimization with MEGA v. 7. The alignments were analysed in Geneious Prime to identify regions with polymorphisms. The design of a set of primers and hydrolysis probes was conducted for each gene alignment using the online version of Primer 3 software (http://bioinfo.ut.ee/primer3-0.4.0/). The specificity of the designed primers, as well as the absence of primer-dimer formation, was subjected to further testing using Primer-Blast (http://www.ncbi.nlm.nih.gov/tools/primer-blast). The primers and probes were custom-synthesized by Metabion (Planegg, Germany) with the probes labelled using FAM (6-carboxyfluorescein) and BHQ-1 (Black Hole Quencher-1).

### PCR conditions and optimization

The cPCR was performed using 25 µL PCR reactions containing 1µM (0.5 µL) of each primer TrafTef1-F and TrafTef1-R, 2x GoTaq Green Master Mix (Promega, USA) (12.5 µL), 2.5 µL of gDNA. Molecular grade water was added to the reaction up to 25 µL. PCR reactions were performed in a gradient block Biometra thermal cycler (AnalytiK Jena, Germany) using following cycling conditions: initial denaturation at 94 °C for 3 min, followed by 35 cycles of 45 s at 94 °C, 45 s annealing with a gradient temperature run from 56 °C to 64 °C and 1 min at 72 °C and a final extension of 7 min at 72 °C. No template control (NTC) consisting of ddH2O was included to check for the absence of contamination. PCR products (3 µL) were checked on 1% agarose gel electrophoresis stained with ROTI GelStain (Carl Roth, Germany). A further check on the conformity of the amplicons with regard to sequence length and species identity was achieved by means of sequencing. Thereby, amplicons derived from *T. afroharzianum* isolates CBS 124,620, AP22TRI85 and MRI349 were sequenced as described in^12^, followed by BLAST search in the NCBI GenBank. The newly generated sequences matched those with accession numbers PQ558230 (1,248 bp), PQ558225 (1,258 bp) and PQ558235 (1,250 bp), respectively, previously generated from the above isolates^12^. As just a small part of known sequences, their addition to GenBank was not considered.

The qPCR was carried out in 12.5 µL final volume containing 0.25 µM (0.125 µL) of forward primer TrafRpb2-F and 0.125 µM (0.125 µL) of reverse primer TrafRpb2-R, 0.15 µM (0.19 µL) of probe TrafRpb2-P, 1x iTaq Universal Probes Supermix (6.25 µL) (Bio-Rad Laboratories, Pty, Ltd.) and 1 µL DNA template. Molecular grade water (4.81 µL) was added to obtain the final volume. The cycling conditions were an initial denaturation at 95 °C for 2 min, and 40 cycles of denaturation at 95 °C for 15 s following by annealing with a gradient temperature run from 60 °C to 66 °C for 1 min. Reactions were performed on the StepOnePlus Real-Time PCR System (Applied Biosystems). No template controls (NTC) consisted of ddH2O. The Ct values and standard deviations of all samples were recorded using the StepOnePlus Software v2.3.

### Construction of a plasmid positive control

The PCR amplicons obtained with primer pairs TrafRpb2q-F/ TrafRpb2q-R (qPCR, 138 bp) and TrafTef1c-F/ TrafTef1c-R (cPCR, 217 bp) from the gDNA of *T. afroharzianum* type strain CBS 124620 were used to generate the plasmid positive control using the pGEM-T Vector System I (Promega, USA) according to the manufacturer’s instructions. The amplicons were cloned with the pGEM-T vector and the competent JM109 cells of Escherichia coli. After transformation, the competent cells were resuspended and transferred on the plates containing LB-Amp (100 µg/ml) – IPTG (40 µg/ml)-x-Gal (40 µg/ml), following by incubation overnight at 37 °C. Liquid culture of positively tested colony was obtained and plasmids were extracted and purified using the QIAprep Spin Miniprep Kit (QIAGEN. The plasmid DNA (pDNA) was diluted in 1× Tris-EDTA buffer (TE) and stored at -20 °C until use. The dilution series of the plasmid was further tested using TrafRpb2-F/TrafRpb2-R and TrafTef1c-F/ TrafTef1c-R to determine the limit of detection (LOD) concentration.

### Validation of the PCR methods

The performance of the PCR assays to amplify *T. afroharzianum* was evaluated by validating different parameters. All DNA extracts were standardized to a concentration of 1 ng µL^− 1^. The analytical specificity was assessed using isolate composition including as non-target 37 of other *Trichoderma* species from maize plants, soil from maize fields and other sources and 14 of non-*Trichoderma* species from maize plants (Table S2). The inclusivity was assessed with a panel of 39 *T. afroharzianum* isolates from different sources (predominantly from maize cobs and soil from maize fields, mycoparasite, Biostimulant products) and geographical origin (Germany, France, Italy, Peru) (Table 1). All assays were conducted with three replicates of each isolate. The pDNA of *T. afroharzianum* strain CBS 124,620 served as positive control and the NTC consisting of ddH2O was included.

The analytical sensitivity was evaluated through a ten-fold serial dilution of pDNA in TE buffer and of pDNA pooled with maize kernel DNA at 1:1 ratio from 1 ng µL^− 1^ concentration with ten replicates being conducted for each template. The standard curve was generated using the obtained cycle threshold (Ct) values and the efficiency of the assay calculated using the equation E = -1 + 10^[(−1/slope)34^. The limit of detection (LOD) was defined as the lowest concentration that could still be amplified in all replicates. The sensitivity of the assays was further evaluated by testing on asymptomatic artificially infected maize cobs obtained from a greenhouse pathogenicity experiment (as described in^12^). The collection of these asymptomatic infected cobs was carried out prior to the manifestation of first symptoms.

Further criteria were evaluated using templates consisting of the pDNA of *T. afroharzianum* (strain CBS 124620) set at 10×LOD and 100×LOD, 0.1 ng µL^− 1^ of *T. afroharzianum* (strain CBS124620) and *T. afroharzianum* (from sample 159 as natural infected maize kernels), 1 ng µL^− 1^ of *T. asperellum* (AP22TRI100) and *T. brevicompactum* (AP22TRI140). Repeatability and reproducibility were assessed by performing each assay (cPCR and qPCR) with these templates in ten and three replicates, respectively. Reproducibility was also assessed by two different operators repeating the assays on two different qPCR platforms: the StepOnePlus Real-Time PCR System (Applied Biosystems) and qTower3 thermocycler (Analytik Jena, Germany). The robustness of the qPCR assays was assessed in ten replicates using reaction volumes of ± 10% (11.25 µL and 13.75 µL) at ± 2 °C modified hybridisation temperatures (60 °C, 62 °C and 64 °C). For the transferability, the qPCR was performed in ten replicates on two qPCR platforms above, and with four different master mixes: iTaq Universal Probes Supermix (Bio-Rad), Maxima Probe/ROX qPCR Master Mix (ThermoFischer Scientific), Applied Biosystems TaqMan Universal PCR Master Mix (Fischer Scientific) and Takara Premix Ex Taq (Probe qPCR) (TaKaRa, Kusatsu, Japan).

### Data analysis

For the qPCR assay, the StepOnePlus software automatically generated the baseline range, threshold cycle (Ct) values and standard curves. The results were checked to ensure they aligned with the provider’s analysis guidelines. The mean threshold cycle (Ct) values were calculated along with their standard deviations (SD). Precision of the assay was evaluated by computing the coefficient of variation (CV). All statistical calculations were performed using Microsoft Excel.

## Results

### Selection of *T. afroharzianum* specific primers and PCR conditions

The design of primers and probes for both cPCR and qPCR was initially based on TEF1α and RPB2 sequences. The final primer and probe sequences are included in Table 2, and Figure S1 illustrates their positions on the corresponding DNA regions, TEF1α for the cPCR and RPB2 for the qPCR. The few qPCR primer combinations obtained on TEF1α predominantly showed cross-reactivity with many non-target *Trichoderma* species (data not shown), despite optimization efforts. By contrast, several qPCR primer combinations were identified based on the RPB2 region. Initial screening using 3 annealing temperatures (60, 62, 64 °C) suggested selecting the TrafRpb2q-F/ TrafRpb2q-R pair and the corresponding TrafRpb2q-P probe, which successfully amplified all target templates at an annealing temperature of 62 °C. The PCR products obtained at annealing temperature of 62 °C show a single band of the expected size of 138 bp in agarose electrophoresis.

**Table 2:** Details on the primers and probes designed and used in this study for conventional and real-time PCR to target *Trichoderma afroharzianum*.

Primer name	Sequence (5‘ to 3‘)	Locus	Product size (bp)
TrafTef1c-F	TTCAGCGACGCTAACCACTT	TEF1α	217
TrafTef1c-R	TGTTAGCACTGGTCCGCAAT	TEF1α	
TrafRpb2q-F	GAGGAGACGGCCATGATCTG	RPB2	138
TrafRpb2q-R	GTGAGTTGTCGGGTTCGTCT	RPB2	
TrafRpb2q-P	Fam -CGTCTTCAGAAGGCCGGTAT- BHQ1	RPB2	

### Reliability of the PCR assays to detect *T. afroharzianum*

#### Analytical specificity

For the specificity, the selected cPCR primers yielded 100% analytical specificity leading to the amplification of a single 217 bp fragment, for all and only the *T. afroharzianum* isolates. In order to ascertain and extend the inclusivity of the cPCR primers, a series of tests were carried out on a panel of samples consisting of kernels from naturally infected and greenhouse-inoculated maize plants. These tests yielded satisfactory results. Amplicon sequencing further confirmed the suitability of the primers to amplify the TEF1α region of *T. afroharzianum*. Comparatively, the qPCR assay exhibited high analytical specificity of 98% (calculated using n-1/n x 100, with *n* = 51 isolates included), due to a single non-target amplification. Among the four *T. gamsii* isolates included in the analysis, isolate AP24TRI434 was amplified with Ct = 21.16 ± 0.33 comparable to true positives. Despite implementing all possible options to optimize the primer specificity (e.g. varying the annealing temperature), this issue persistently occurred. In addition, the qPCR assay demonstrated 100% inclusivity by amplifying gDNA from all the *T. afroharzianum* isolates (mean Ct = 19.25–25.38, SD = 0.2–1.47; *n* = 10) (Table 1), regardless of the pathogenicity, host and geographical origin.

#### Analytical sensitivity

**Fig. 1:**
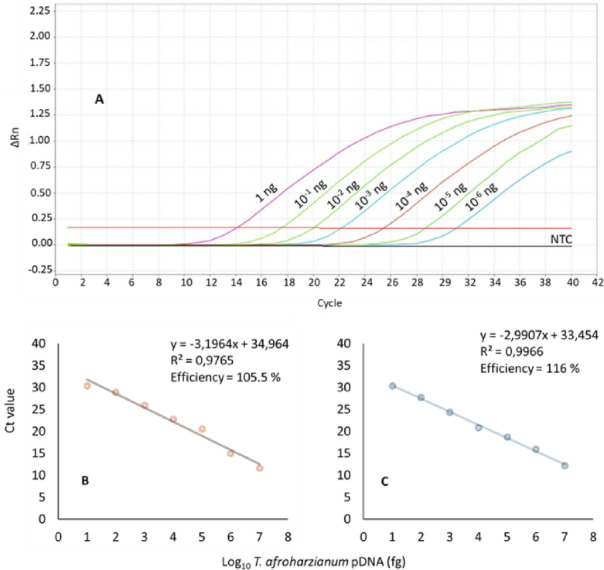
Real-time assay (**A**). Amplification curve of the RPB2 gene using a 10-fold serial dilution of pDNA for determining the limit of detection (LOD, sensitivity) (**B**,**C**). Standard curve assessed with a 10-fold serial dilution of the *T. afroharzianum* (strain CBS 124620) plasmid DNA (pDNA) positive control. Dilution started from 1 ng µL^− 1^ concentration (**B**). pDNA in TE buffer; (**C**). pDNA in 1 ng µL^− 1^ of gDNA from healthy maize kernels. The mean Ct values were calculated with ten replicates.

Analytical sensitivity differed between the species-specific cPCR and qPCR assays. In a tenfold serial dilution of plasmid DNA ranging from 1 ng to 10^− 8^ ng µL⁻¹, the cPCR consistently produced amplicons of the expected size (217 bp) in 100% of replicates (*n* = 10) down to a concentration of 1 pg µL⁻¹ (= 10^− 3^ ng µL⁻¹) using DNA from pure cultures. In contrast, the qPCR consistently amplified target DNA in all replicates (*n* = 10) down to a concentration of 1 fg µL⁻¹ (= 10^− 6^ ng µL⁻¹) (Fig. 1A). Linear calibration curves generated from mean Ct values plotted against the logarithm of template concentration are shown in Fig. 1B–C. Fully repeatable positive results (100%) were obtained down to the determined limit of detection (LOD) of 1 fg µL⁻¹ using plasmid DNA (pDNA) diluted in TE buffer (mean Ct = 30.4) and pDNA diluted in 1 ng µL⁻¹ genomic DNA extracted from healthy maize kernels (mean Ct = 30.8). For pDNA diluted in TE buffer, the standard curve showed a correlation coefficient (R²) of 0.977, a slope of − 3.20, and an amplification efficiency of 105.5% (Fig. 1B). When pDNA was diluted in genomic DNA from healthy maize kernels, the corresponding values were R² = 0.997, a slope of − 2.99, and an efficiency of 116% (Fig. 1C). Both cPCR and qPCR assays successfully detected *T. afroharzianum* in all samples derived from maize kernels collected from asymptomatic infected cobs. The qPCR assay yielded Ct values ranging from 27 to 29, indicating its suitability for detecting *T. afroharzianum* in asymptomatic samples at concentrations close to the determined LOD.

#### Further performances values

Both assays demonstrated satisfactory repeatability, reproducibility, robustness, and transferability when evaluated using a panel comprising plasmid target DNA and genomic DNA from pure cultures as well as naturally infected samples. Both the cPCR and qPCR assays amplified exclusively templates containing target DNA, whereas no amplification was observed for non-target DNA in any replicate, resulting in 100% repeatable and reproducible outcomes (Supplementary Table S3 for statistical data to qPCR). The coefficients of variation (CVs) ranged from 0.44% to 1.05% for repeatability and from 0.48% to 0.72% for reproducibility with the qPCR assay, which also exhibited high robustness with respect to variations in experimental parameters. Alterations in reaction volume (12.5 µL) or hybridisation temperature (60 °C) had little effect on assay performance. However, a slight decrease in Ct values was observed at an annealing temperature of 64 °C (Supplementary Table S4). Furthermore, analysis of the template panel by two independent operators yielded the expected results for both target and non-target templates. These results were consistent across operators and showed very low variation in Ct values, confirming the repeatability and reproducibility of the assay (data not shown).

The cPCR demonstrated 100% transferability when tested on Applied Biosystems, Biometra and Bio-Rad Thermocyclers with 2x GoTaq Green Master Mix (Promega, USA) and Roche Taq DNA Polymerase (Roche, Germany). The transferability of the qPCR assay was evaluated using three different commercial master mixes and an alternative PCR platform. Changes in the master mix or PCR platform did not affect assay specificity, in addition the repeatability and reproducibility were maintained across all tested conditions (Supplementary Table S5). The observed differences were primarily related to assay sensitivity. When using the StepOnePlus system, the TaKaRa Premix Ex Taq (Probe qPCR) produced Ct values comparable to those obtained with the iTaq Universal Probes Supermix (Bio-Rad). The Maxima Probe/ROX qPCR Master Mix increased assay sensitivity, resulting in Ct values that were 0.67–2.04 cycles lower than those obtained with the iTaq Universal Probes Supermix. In contrast, use of the iTaq Universal Probes Supermix on the qTower3 thermocycler (Analytik, Jena) resulted in reduced robustness, with coefficients of variation ranging from 2.90% to 3.32%, and Ct values that were 1.25–3.24 cycles higher than those obtained using the StepOnePlus system.

## Discussion

Trichoderma-associated diseases in maize production are an increasing concern. The threat to this key cereal crop is likely to intensify due to the widespread occurrence and persistence of *Trichoderma* species in agricultural soils worldwide^19,35–37^. Moreover, *Trichoderma* species are widely used as biological control agents, and the pathogenic potential of certain species or strains therefore represents a significant challenge for the safe application, regulation, and acceptance of *Trichoderma*-based biocontrol products. Accordingly, the development of molecular tools for the accurate identification of pathogenic *Trichoderma* species is essential for improving the understanding and management of these diseases. Here, we provide a PCR-based tool for the rapid and sensitive detection of *T. afroharzianum*. Current knowledge indicates that multiple *Trichoderma* species are associated with diseases in maize^12,15,16^, therefore, the implementation of multiplex PCR assays enabling the simultaneous detection of multiple pathogens represents a rational and efficient diagnostic approach^38^. However, epidemiological data from Europe indicate that *T. afroharzianum* has consistently been identified as the predominant etiological agent of Trichoderma ear rot^11,13^. Under these circumstances, species-specific detection of this pathogen using simplex PCR remains justified.

The most significant achievement of this study was the development of a diagnostic tool based on complementary cPCR and qPCR assays. Despite the longer processing time associated with cPCR, it remains a cost-effective option compared with the more expensive qPCR approach^39^, which in turn provides the advantage of higher sensitivity^40^. The selection of target genes for primer design was guided by established molecular identification practices for *Trichoderma* species, which rely primarily on the ITS, TEF1α, and RPB2 regions for phylogenetic analysis and DNA barcoding^19^. For PCR-based detection, ITS markers are generally effective at the genus level^41–43^, whereas TEF1α and RPB2 are more suitable for species-specific discrimination^19,28,44–46^. Both TEF1α and RPB2 have previously been demonstrated to reliably target *T. afroharzianum*^37,45,46^. In the present study, TEF1α was identified as the most suitable gene for cPCR, whereas RPB2 was found to be more appropriate for the qPCR assay.

A previous study developed a qPCR assay for the detection and quantification of *T. afroharzianum* causing maize ear rot in Italy^46^. That assay targeted the TEF1α gene, based on the assumption that conserved single-nucleotide polymorphisms within this region allow discrimination of *T. afroharzianum* from other species within the Harzianum clade. However, the qPCR primers designed in the same TEF1α region as in study^46^ exhibited unexpected cross-reactivity with certain *T. asperellum* and *T. atrobrunneum* isolates. Higher specificity for *T. afroharzianum* was only achieved when primers targeting the RPB2 gene were employed. This single-copy and highly conserved RPB2 gene plays a central role in resolving the phylogeny of *Trichoderma* species^12,19^ and has increasingly been recognised as a suitable and reliable marker for PCR-based detection of *Trichoderma* species^38,44^. Regarding real-time PCR chemistry, SYBR Green used by^46^ has been widely applied for the detection and quantification of *Trichoderma* species, particularly in studies addressing their biocontrol potential^47,48^. The TaqMan PCR system is likewise well established for diagnostic and quantitative applications^45,49,50^ and was selected in the present study for two main reasons: first, to provide a user-friendly diagnostic tool, and second, because of its superior accuracy and specificity^51^.

A rigorous validation process demonstrated that the proposed assays largely fulfil the requirements for new diagnostic methods as defined by^32^. Both assays exhibited high specificity against the tested *Trichoderma* species and other fungi associated with maize cobs and a range of additional host plants. Although cross-reactivity of the qPCR assay was observed with the *T. gamsii* isolate AP14TRI434, this isolate originated from soil and was shown to be non-pathogenic to maize. Consequently, it can be inferred that neither the cPCR nor the qPCR assay amplifies *Trichoderma* isolates capable of causing field or greenhouse symptoms comparable to those induced by *T. afroharzianum*^12,15^. Furthermore, the case of false positive with qPCR can still be revealed by applying the cPCR. The complementarity of both assays was demonstrated in this case. Both assays achieved 100% inclusivity for all *T. afroharzianum* isolates tested, encompassing a broad range of geographic origins, host associations, and pathogenicity profiles^12^. It is important to note that not all *T. afroharzianum* strains are pathogenic to maize. Of the 39 *T. afroharzianum* isolates obtained during the survey for this study and tested on maize, all 33 from corn cobs (both with and without ear rot symptoms) and soil from corn fields were found to be pathogenic. In contrast, the 6 non-pathogenic isolates were from alternative sources, such as mycoparasite, endophyte, or Biostimulant. This observation could be significant in relation to the current interpretation of the diagnostic result.

Presently, the factors determining the lifestyle of *T. afroharzianum* isolates remain to be elucidated. Furthermore, there is an absence of reliable molecular markers with which to differentiate between these isolates. Phylogenetic analyses^12^ and PCR assays (this study) have thus far proven incapable of distinguishing between pathogenic and non-pathogenic *T. afroharzianum* isolates. The subsequent stage in resolving this issue is a genomic analysis that draws upon the findings of preceding studies^52,53^. A previous study of *Phyllosticta* spp. on citrus revealed several genomic differences between species with different lifestyles, finding that the carbohydrate-active enzyme profile of a species correlated with its trophic state^53^. At present, the aforementioned knowledge deficiency directly affects the interpretation and perception of the diagnostic results of *T. afroharzianum*. Consequently, in the event of a positive diagnostic result from latent infections, it is imperative that pathogenicity testing is conducted in order to confirm that the detected *T. afroharzianum* isolate is capable of causing disease. Conversely, maize samples exhibiting characteristic Trichoderma ear rot symptoms but yielding negative results in these assays should be further evaluated for the presence of other *Trichoderma* species that may also be pathogenic to maize.

The high sensitivity of the qPCR assay is consistent with previous assays targeting *Trichoderma* species^44,54^. The LOD of 1 fg µL⁻¹ determined for the TaqMan assay represents a substantial improvement over the LOD of 50 fg µL⁻¹ reported for a SYBR Green based assay^46^. This pronounced difference in sensitivity is unlikely to be attributable solely to the detection chemistry and is more likely related to differences in sample preparation or reaction composition^55^. The presence of host plant material had no detectable effect on analytical sensitivity, as indicated by comparable results obtained from pure cultures, naturally infected samples, and artificially inoculated material. Although minor inhibitory effects caused by co-extracted DNA cannot be entirely excluded^54,56^, such effects are unlikely to compromise assay performance when testing maize kernels. Concerning the higher qPCR efficiency of 116% obtained with gDNA diluted in maize DNA, it may be attributable to the presence of PCR inhibitors in the maize DNA, dilution artefacts or standard curve slope distortion. However, this does not compromise the validity of the assay performance as despite this elevated calculated efficiency, linearity (R² ≥ 0.996) and repeatability remained within EPPO acceptance criteria.

Both assays successfully detected *T. afroharzianum* in asymptomatic material at early infection stages (7–10 days post inoculation), underscoring their suitability for early diagnosis. Furthermore, while the assays were validated exclusively on maize material, and the manuscript is based on this, they were nevertheless tested and found to be reliable in detecting *T. afroharzianum* in soil as well. It is evident from the epidemiological observations^11^ that *T. afroharzianum* is capable of infecting maize from the soil. Therefore, the assays can be utilised for monitoring the presence of *T. afroharzianum* in the soil of maize fields, and for the prediction of the risk of Trichoderma ear rot. As expected, both assays demonstrated high repeatability, reproducibility, and robustness. While the cPCR assay was readily transferable across platforms, the qPCR assay requires careful verification prior to diagnostic implementation, particularly when using platforms other than the StepOnePlus system. For example, testing on the qTower qPCR device resulted in delayed amplification signals, which could lead to false-negative results in samples containing low levels of target DNA, a limitation that is especially relevant for latent infections. However, the risk of false negatives in symptomatic samples is considered low, as maize tissues affected by Trichoderma ear rot typically exhibit extensive fungal colonization^12,13,15^, providing sufficiently high concentrations of target DNA.

## Conclusion

The aim of this study was to facilitate the diagnosis of the newly emerging Trichoderma ear rot by providing a rapid and efficient molecular tool. The two PCR assays developed and validated here are well suited to achieve this objective. A key advantage of this approach is the use of two complementary PCR systems endpoint and TaqMan real-time PCR targeting different genetic loci. This tool enables the rapid detection of *T. afroharzianum* directly from infected maize tissue, thereby eliminating the need for culture-based isolation. Furthermore, the high sensitivity of the qPCR assay allows the detection of latent infections, which is essential for early diagnosis. However, given that not all *T. afroharzianum* strains are capable of causing disease, the detection of this species in asymptomatic cobs does not constitute evidence of ear rot. Indeed, in field conditions, *T. afroharzianum* can only be confirmed as the causal agent of ear rot when disease symptoms are present or following pathogenicity testing. The ability to identify *T. afroharzianum* ear rot at early stages is critical for the timely implementation of management strategies aimed at minimizing yield losses and preventing further spread of the disease.

## Supplementary Information

Supplementary Information.

## Data Availability

Data on the development and validation of the PCR tests can be found in the main manuscript and the supplemental material (Tables S2–5). Details of the DNA sequences used to identify the Trichoderma isolates were referenced in previous work^12^. They are provided in supplementary material (Table S1) and are also available in the NCBI database (https://www.ncbi.nlm.nih.gov/nucleotide? cmd=search).
