# Supplementary material for: Development and validation of conventional and TaqMan real-time PCR for the detection of Trichoderma afroharzianum causing corn ear rot
Source: Sci Rep. 2026 May 6;16:14427. doi: 10.1038/s41598-026-51199-2 (PMC13149868; doi:10.1038/s41598-026-51199-2)
Supplement: Supplementary file 1 — Supplementary Information. [file 41598_2026_51199_MOESM1_ESM.pdf]

Development and validation of conventional and TaqMan real-time PCR for the detection of *Trichoderma afroharzianum* causing corn ear rot

Clovis Douanla-Meli[1]*, Annette Pfordt[2], Andreas von Tiedemann[2], Gritta Schrader[1], Bernhard Carl Schäfer[1]
[1] Julius Kühn-Institut (JKI) - Federal Research Centre for Cultivated Plants, Institute for National and International Plant Health, Germany
[2] Plant Pathology and Crop Protection, Georg-August University of Göttingen, Göttingen, Ger-many

*Corresponding author: Clovis Douanla-Meli (Clovis.Douanla-Meli@julius-kuehn.de)
https://orcid.org/0000-0001-7182-5905

Supplementary information

Figure S1. Alignment of representative sequences of *Trichoderma afroharzianum* and other *Trichoderma* species of the RPB2 gene (A) for the real-time PCR and TEF1α gene (B) for the endpoint PCR. Primers and hydrolysis probe are flagged in blue for forward primer, in green for the reverse primer and in red for the probe.

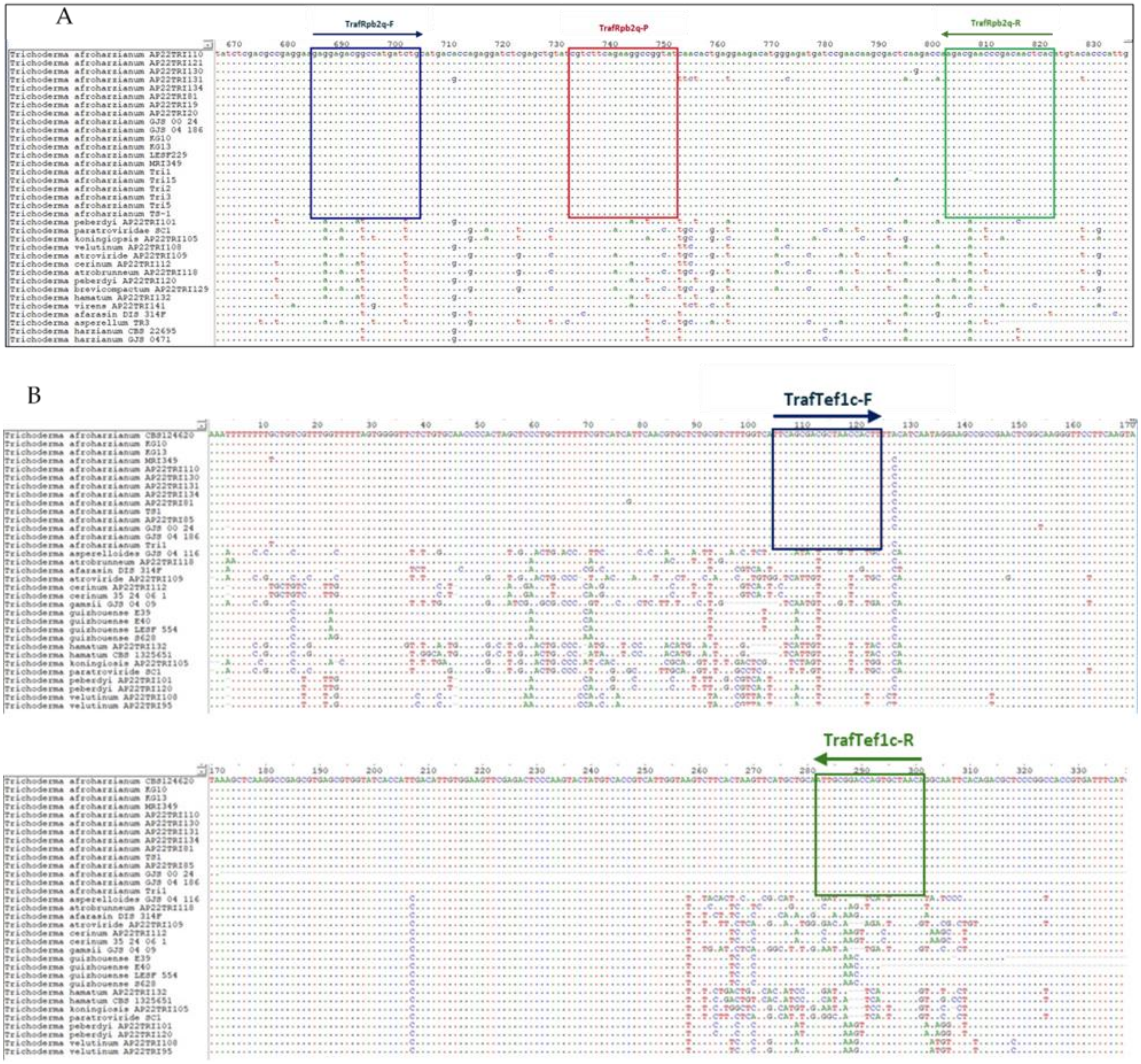

Table S1. Details on the sequences used in the phylogeny to identify the *Trichoderma* strains that were used to develop the PCR tests. Sequences with GenBank accession numbers PQ558164–PQ558272 were newly generated during the TricoMais project, which focuses on the new Trichoderma ear rot.

**Species**	**Code**	**Year**	**Geographic origin**	**Host/substrat**	**GenBank**	
					***TEF1*-α**	***RPB2***
T. afarasin	DIS 377A	-	Cameroon	Stem, endophyte	FJ463322	FJ442799
T. afarasin	Dis 314F	-	Cameroon	Stem, endophyte	FJ463400	FJ442778
T. afroharzianum	AP18TRI1	2018	Croix de Pardies (FR)	Maize	PQ558165	PQ629815
T. afroharzianum	AP18TRI2	2018	Kuenzing (DE)	Maize	PQ558166	PQ629816
T. afroharzianum	AP18TRI3	2018	Pocking (DE)	Maize	PQ558167	PQ629817
T. afroharzianum	AP19TRI5	2019	Bernburg (DE)	Maize	PQ558170	PQ629820
T. afroharzianum	CBS 124620	-	-	-	PQ558230	PQ629874
T. afroharzianum	KG10	2018	Kavadarci (MKD)	*P. ostreatus*	PQ558233	PQ629877
T. afroharzianum	KG13	2018	Kavadarci (MKD)	*P. ostreatus*	PQ558234	PQ629878
T. afroharzianum	MRI349	-	-	-	PQ558235	PQ629879
T. afroharzianum	DISAFATS-1	2021	Carmagnola (IT)	Maize	PQ558263	PQ629907
T. afroharzianum	AP22TRI81_1	2022	Rustenhart (DE)	Maize	PQ558223	PQ629867
T. afroharzianum	AP22TRI84	2022	Rustenhart (DE)	Maize	PQ558224	PQ629868
T. afroharzianum	AP22TRI85	2022	Rustenhart (DE)	Maize	PQ558225	PQ629869
T. afroharzianum	AP22TRI99	2022	Bevern (DE)	Soil	PQ558267	PQ629911
T. afroharzianum	AP22TRI110	2022	Hasloch 1 (DE)	Soil	PQ558183	PQ629833
T. afroharzianum	AP22TRI111	2022	Hasloch 2 (DE)	Soil	PQ558268	PQ629912
T. afroharzianum	AP22TRI113	2022	Bevern Hoeltje (DE)	Soil	PQ558185	PQ629835
T. afroharzianum	AP22TRI114	2022	Bevern Ricke (DE)	Soil	PQ558186	PQ629836
T. afroharzianum	AP22TRI130	2022	Oberpframmern (DE)	Soil	PQ558201	PQ629848

T. afroharzianum	AP22TRI131	2022	Oberpframmern (DE)	Soil	PQ558202	PQ629849
T. afroharzianum	AP22TRI134	2022	Oberpframmern (DE)	Soil	PQ558205	PQ629850
T. afroharzianum	AP22TRI136	2022	Oberpframmern (DE)	Soil	PQ558207	PQ629852
T. afroharzianum	AP22TRI137	2022	Rustenhart (DE)	Soil	PQ558208	PQ629853
T. afroharzianum	AP22TRI148	2022	Haßloch 2 (DE)	Soil	PQ558269	PQ629913
T. afroharzianum^{T}	GJS 04 186	-	Peru	*Moniliophthora roreri*	FJ463301	FJ442691
T. afroharzianum	LESF229	-	-	Soil	KT279013	KT278945
T. afroharzianum	Tri-1	-	-	-	OP102131	OP102132
T. arundinaceum	TR1	2016	Leskovac (SRB)	Tomato, root	-	PQ629917
T. arundinaceum	AP22TRI129	2022	Nossen (DE)	Soil	PQ558200	-
T. arundinaceum	ATCC 90237	-	Namibia	Soil	EU338291	EU338326
T. arundinaceum	GJS 05-183	-	Iran	-	EU338274	EU338302
T. asperelloides	**HOHTR22**	-	-	-	PQ558256	PQ629900
T. asperelloides	GJS 04-116	-	Vietnam	Soil	GU248412	GU248411
T. asperelloides	NT8	-	Nepal	Soil	MW408215	MZ355338
T. asperellum	TR2	2015	Topola (SRB)	Apricot, fruit	PQ558255	PQ629899
T. asperellum	TR4	2015	Topola (SRB)	Apricot, fruit	PQ558266	PQ629910
T. asperellum	XILONT34	-		-	PQ558265	PQ629909
T. asperellum	ABITEP02	-		-	PQ558164	PQ629814
T. asperellum	AP22TRI100	2022	Bevern Forst (DE)	Soil	PQ558173	PQ629823
T. asperellum	AP22TRI142	2022	Rustenhart (DE)	Soil	PQ629875	PQ629922
T. asperellum	CGMCC 6422	-	China	-	KF425756	KF425755
T. asperellum	GJS 06-314	-	Ecuador	Plantain	GU198292	GU198278
T. atrobrunneum	T54	2008	Kula (SRB)	*A. bisporus* fb	PQ558272	PQ629914
T. atrobrunneum	AP22TRI149	2022	Bevern Höltje (DE)	Soil	PQ558218	PQ629862
T. atrobrunneum	GJS 04-67	-	Italy	Soil	FJ463360	FJ442724
T. atrobrunneum	SZMC 26673	-	North Macedonia	*P. ostreatus*	MZ773434	MZ773413
T. atroviride	IPP0316	1976	-	Food	PQ558231	

T. atroviride	T33	2006	Zemun Polje (SRB)	*A. bisporus fb*	PQ558243	PQ629887
T. atroviride	T60	2008	Zemun (SRB)	*A. bisporus fb*	PQ558250	PQ629894
T. atroviride	TR8	2019	Belgrade (SRB)	Tomato, root	PQ558261	PQ629905
T. atroviride	TR10	2019	Leskovac (SRB)	Tomato, rhizosphere	PQ558253	PQ629897
T. atroviride	HOHT20	-	-	-	PQ558240	PQ629884
T. atroviride	AP22TRI102	2022	Bevern Lindenbusch (DE)	Soil	PQ558175	PQ629825
T. atroviride	AP22TRI103	2022	Göttingen Königsbühl (DE)	Soil	PQ558176	PQ629826
T. atroviride	AP22TRI107	2022	Pocking (DE)	Soil	PQ558180	PQ629830
T. atroviride	AP22TRI109	2022	Pocking (DE)	Soil	PQ558182	PQ629832
T. atroviride	AP22TRI115	2022	Granswang KGW 3 (DE)	Soil	PQ558187	PQ629837
T. atroviride	AP22TRI116	2022	Granswang KGW 3 (DE)	Soil	PQ558188	PQ629838
T. atroviride	AP22TRI117	2022	Granswang KGW 3 (DE)	Soil	PQ558189	-
T. atroviride	AP22TRI119	2022	Granswang KGW 3 (DE)	Soil	PQ558191	PQ629840
T. atroviride	AP22TRI128	2022	Kuenzing (DE)	Soil	PQ558199	PQ629847
T. atroviride	AP22TRI139	2022	Rustenhart (DE)	Soil	PQ558210	PQ629855
T. atroviride	CBS 119499	-	-	-	FJ860611	FJ860518
T. atroviride	CBS 141.95	2003	Slovenia	Beetle gallery	AY376051	EU341801
T. auriculariae[T]	JZBQT1Z7	-	China	-	ON649896	ON649949
T. auriculariae	JZBQT1Z8	-	China	-	ON649897	ON649950
T. azevodoi	IPP0318	1992	Ban Vieng (CN)	Soil	PQ629921	PQ629920
T. azevodoi	IPP0320	1992	Chiang Mai (CN)	Soil	PQ558271	PQ629876
T. azevodoi	AP19TRI6	2019	KWS (DE)	Maize	PQ558171	PQ629923
T. azevodoi	AP19TRI7	2019	Grucking (DE)	Maize	-	PQ629915
T. azedevoi	AP19TRI8	2019	Großumstadt (DE)	Maize	PQ558172	PQ629822
T. azedevoi	AP19TRI10	2019	Großumstadt (DE)	Maize	PQ558168	PQ629818

T. azevedoi	AP19TRI11	2019	Pfaffenhofen (DE)	Maize	PQ558169	PQ629819
T. azevodoi	CEN1403	-	Brazil	Soil	MK696638	MK696800
T. azevodoi	CEN1422	-	Brazil	Soil	MK696660	MK696821
T. azevodoi	CEN1423	-	Brazil	Soil	MK696661	MK696822
T. bannaense[T]	HMAS 248840	-	China	-	KY688037	KY687979
T. bannaense	HMAS 248865	-	China	-	KY688038	KY688003
T. breve[T]	HMAS 248844	-	China	-	KY688045	KY687983
T. breve	HMAS 248845	-	China	-	KY688046	KY687984
T. brevicompactum	AP22TRI140	2022	Rustenhart (DE)	Soil	PQ558211	-
T. brevicompactum	CBS 112444	-	Mexico	Soil	EU338296	EU338314
T. brevicompactum	CBS 112447	-	Mexico	Soil	EU338300	EU338318
T. camerunense	Vimi-17 0025	-	Brazil	Termite nest	MZ675902	MZ675866
T. camerunense	Vimi-17 0034	-	Brazil	Termite nest	MZ675908	MZ675862
T. cerinum	AP22TRI97	2022	Göttingen (DE)	Soil	PQ558228	PQ629872
T. cerinum	AP22TRI112	2022	Hasloch 3 (DE)	Soil	PQ558184	PQ629834
T. cerinum	AP22TRI126	2022	Nossen (DE)	Soil	PQ558197	PQ629846
T. cerinum	AP22TRI138	2022	Rustenhart (DE)	Soil	PQ558209	PQ629854
T. cerinum	AP22TRI150	2022	Bevern Höltje (DE)	Soil	PQ558219	PQ629863
T. cerinum	31.24.06.1	-	Poland	-	KP008913	KP009174
T. cerinum	S357	-	France	-	KF134797	KF134788
T. endophyticum	Dis 221E	-	Ecuador	*Theobroma gileri*	FJ463316	FJ442775
T. endophyticum	MMSRG85	-	Brazil	*Botrylloides giganteus*	OQ291284	OQ291285
T. guizhouense	T10	2006	Požarevac (SRB)	*A. bisporus* fruiting body (fb)	KC555182	PQ629882
T. guizhouense	T52	2008	Zemun (SRB)	*A. bisporus fb*	KC555177	PQ629890
T. guizhouense	T57	2008	Ugrinovci (SRB)	*L. edodes fb*	MT876593]	PQ629891
T. guizhouense	T59	2008	Ugrinovci (SRB)	*L. edodes fb*	MT876595	PQ629893
T. guizhouense	T2	2017	Kruševac (SRB)	Strawberry	PQ558239	PQ629883
T. guizhouense	T3	2017	Leskovac (SRB)	Tomato, rhizosphere	PQ558242	PQ629886

T. guizhouense	T4	2017	Čelarevo (SRB)	Cherry, rhizosphere	PQ558245	PQ629889
T. guizhouense	TR5	2017	Užice (SRB)	Tomato, rhizosphere	PQ558258	PQ629902
T. guizhouense	TRICHOSTAR T58	-		Biostimulant	PQ558248	PQ629892
T. guizhouense	E39	-	Kenya	*Coffea* sp.	MK044084	MK044176
T. guizhouense	S548	-	Spain	-	KJ665507	KJ665031
T. guizhouense	S628	-	Greece	-	KJ665511	KJ665273
T. guizhouense	SZMC 22514	-	Croatia	*P. ostreatus*	MZ773448	MZ773427
T. guizhouense	DIS 314D	-	Cameroon	Stem	FJ463355	FJ442719
T. hamatum	AP22TRI152	2022	Oberpframmern (DE)	Soil	PQ558221	PQ629865
T. hamatum	CBS:132565		France		KJ665514	KJ665275
T. hamatum	Dis 216d	2022	Ecuador	Soil	EU856313	FJ150778
T. harzianum	T1	2017	Kruševac (SRB)	Strawberry	PQ558237	PQ629881
T. harzianum	TR7	2016	Požega (SRB)	Tomato, rhizosphere	PQ558260	PQ629904
T. harzianum	AP22TRI104	2022	Göttingen Königsbühl (DE)	Soil	PQ558177	PQ629827
T. harzianum	AP22TRI106	2022	Pocking (DE)	Soil	PQ558179	PQ629829
T. harzianum	AP22TRI123	2022	Granswang KGW 4 (DE)	Soil	PQ558195	PQ629844
T. harzianum	AP22TRI135	2022	Oberpframmern (DE)	Soil	PQ558206	PQ629851
T. harzianum	AP22TRI151	2022	Oberpframmern (DE)	Soil	PQ558220	PQ629864
T. harzianum	AP22TRI153	2022	Bevern Ricke (DE)	Soil	PQ558222	PQ629866
T. harzianum	CBS 226.95[N]	-	-	-	MH874152	AF545549
T. harzianum	DIS 314D	-	Cameroon	Stem	FJ463355	FJ442719
T. harzianum	GJS 04-71	-	Italy	*Castanea sativa*	FJ463396	FJ442779
T. harzianum	GJS 05-107	-	Italy	*Ricinus communis*	FJ463329	FJ442708
T. inhamatum[T]	CBS 273.78	-	Colombia	Soil maize field	AF348099	FJ442725

T. koningii	T39	2007	Veliko Gradište (SRB)	*A. bisporus fb*	PQ558244	PQ629888
T. koningii	GJS 90-18		USA:WI	Burned wood	DQ289007	EU248600
T. koningiopsis	AP22TRI98	2022	Göttingen (DE)	Soil	PQ558229	PQ629873
T. koningiopsis	AP22TRI105	2022	Göttingen (DE)	Soil	PQ558178	PQ629828
T. koningiopsis	GJS 93-20	-	Cuba	Branch	DQ284966	EU241506
T. lentiforme	DIS 218E	2003	Ecuador	*Theobroma gileri*	FJ463310	FJ442793
T. lentiforme	DIS 173F	2003	Brazil	*Theobroma* sp.	FJ463347	FJ442787
T. linzhiense	HMAS 248874	2016	China, Tibet	Soil	KY688048	KY688011
T. linzhiense[T]	HMAS 248846	2015	China, Tibet	Soil	KY688047	KY687985
T. neocrassum[T]	DAOM 164916	2014	Canada	*Picea* sp. wood	EU80048	KJ842185
T. neocrassum	GJS 95-157	-	New York, USA	Decorticated wood	AF534602	AF545543
T. paratroviride	VINTEC_SC1	-	-	-	PQ558236	PQ629880
T. paratroviride	a131	2022	China	Maize	ON934351	ON934386
T. paratroviride[T]	CBS:136489	2010	Spain	*Phillyrea angulistifolia*	KJ665627	KJ665321
T. paraviridescens	HOHUHBot	-	-	-	PQ558264	PQ629908
T. paraviridescens	CBS 274.79	-	Austria	-	DQ307513	EU252010
T. paraviridescens	S122	-	Italy	-	KC285671	KC285764
T. peberdyi	AP22TRI96	2022	Bevern (DE)	Soil	PQ558227	PQ629871
T. peberdyi	AP22TRI101	2022	Bevern Forst (DE)	Soil	PQ558174	PQ629824
T. peberdyi	AP22TRI120	2022	Granswang (DE)	Soil	PQ558192	PQ629841
T. peberdyi	AP22TRI122	2022	Granswang (DE)	Soil	PQ558194	PQ629843
T. peberdyi	AP22TRI124	2022	Granswang (DE)	Soil	PQ558196	PQ629845
T. peberdyi	AP22TRI143	2022	Haßloch 1 (DE)	Soil	PQ558213	PQ629857
T. peberdyi	AP22TRI144	2022	Haßloch 1 (DE)	Soil	PQ558214	PQ629858
T. peberdyi	AP22TRI145	2022	Haßloch 1 (DE)	Soil	PQ558215	PQ629859
T. peberdyi	AP22TRI146	2022	Haßloch 1 (DE)	Soil	PQ558216	PQ629860
T. peberdyi	AP22TRI147	2022	Haßloch 2 (DE)	Soil	PQ558217	PQ629861
T. peberdyi	CEN1387	2015	Brazil	Soil	MK696619	MK696781
T. peberdyi	CEN1388	2015	Bzazil	Soil	MK696620	MK696782
T. petersenii	CBS 119507	2003	Austria	*Salix caprea*	FJ860670	FJ860568

T. petersenii	GJS 04-164	-	Tennessee, USA	Decorticated wood	DQ289004	FJ442783
T. polypori[T]	HMAS 248855	2015	China, Hunan	Dried polypore	KY688058	KY687994
T. polypori	HMAS 248861	2015	China, Hunan	Polypore	KY688059	KY688000
T. protrudens	DIS 119F	-	India	*Theobroma cacao*	EU338289	EU338322
T. protrudensi[T]	CBS 121320	-	India	*Theobroma cacao*	-	OK813901
T. pseudopyramidale[T]	E720	-	Ethiopia	*Coffea arabica*	MK044131	MK044224
T. pseudopyramidale	M307	-	Ethiopia	*Coffea arabica*	MK044162	MK044225
T. pyramidale[T]	CBS 135574	-	Italy	-	KJ665699	KJ665334
T. pyramidale	S573	-	Italy	-	KJ665698	-
T. rifaii	Dis 337F	-	Panama	Stem, endophyte	FJ463321	FJ442720
T. rifaii[T]	DIS 355B	-	Ecuador	*Theobroma gileri*	FJ463324	-
T. simmonsii	T64	2009	Zemun (SRB)	*A. bisporus fb*	PQ558251	PQ629895
T. simmonsii	TR9	2018	Leskovac (SRB)	Tomato, rhizosphere	PQ558262	PQ629906
T. simmonsii	BIOHEALTH_T50	-	-	-	PQ629919	PQ629918
T. simmonsii	GJS 90-127	-	North Carolina, USA	Wood and fungus	-	FJ442798
T. simmonsii	S7	-	Italy	-	KJ665719	KJ665337
T. simmonsii	SZMC 26671	-	Serbia	*Pleurotus ostreatus*	MZ773436	MZ773415
Trichoderma sp1.	TR6	2010	Belgrade (SRB)	Orchids, rhizosphere	PQ558259	PQ629903
Trichoderma sp1.	TR11	2011	Belgrade (SRB)	Orchids, leaf	PQ558254	PQ629898
Trichoderma sp2.	TERRAX_T720	-	-	-	PQ558252	PQ629896
Trichoderma sp2.	AP22TRI118	2022	Granswang (DE)	Soil	PQ558190	PQ629839
T. tomentosum	CBS 120637	-	-	-	FJ860629	FJ860532
T. tomentosum[T]	DAOM 178713A	-	-	-	EU279969	AF545557
T. velutinum	AP22TRI95	2022	Bevern (DE)	Soil	PQ558226	PQ629870
T. velutinum	AP22TRI108	2022	Pocking (DE)	Soil	PQ558181	PQ629831

T. velutinum	AP22TRI121	2022	Granswang (DE)	Soil	PQ558193	PQ629842
T. velutinum	CPK 298	-	Nepal	-	KF134794	KJ665769
T. velutinum^{T}	DAOM 230013	-	Nepal	-	AY605803	JN133569
T. virens	AP22TRI141	2022	Rustenhart (DE)	Soil	PQ558212	PQ629856
T. virens	DIS 162	-	Costa Rica	*Theobroma cacao*	FJ463367	FJ442696
T. virens	E174	-	Cameroon	*Coffea brevipes*	MK044087	MK044180
T. virens	GJS 01-287	-	Cote d'Ivoire	Soil	AY750894	EU341804

Table S2. Fungal isolates used in this study for assessing the specificity of the conventional and real-time PCR assays. For each isolate following details are provided: taxonomy, name, year of collection, source or host, location and the results of testing with endpoint and real-time PCR. The pathogenicity of all *Trichoderma* isolates on maize was tested in previous work (Pfordt et al., 2025) in a greenhouse (see legend below).

Species	Isolate	Year	Source/Host	Location	Assay (target)	
					cPCR	qPCR
T. asperelloides^{NP}	HOHTR22		-	University Hohenheim	-	>40
T. asperellum ^{P*}	TR4		Apricot, fruit	Serbia	-	>40
T. asperellum^{P}	XILONT34		Biostimulant	Kwizda Agro GmbH	-	>40
T. asperellum^{P}	ABITEP02		ABiTEP GmbH Berlin	Biostimulant	-	>40
T. asperellum ^{P*}	AP22TRI100	2022	Soil maize field	Germany	-	>40
T. atrobrunneum^{NP}	AP22TRI118	2022	Soil maize field	Germany	-	>40
T. atrobrunneum^{NP}	T54		*A. bisporus* fruitbodies	Serbia	-	>40
T. atroviride^{NP}	IPP0316	1976	Infant food	-	-	>40
T. atroviride^{NP}	T33		*A. bisporus* fruitbodies	Serbia	-	>40
T. atroviride^{P*}	T60	2008	*A. bisporus* fruitbodies	Serbia	-	>40
T. atroviride^{NP}	TR10	2019	Tomato, rhizosphere	Serbia	-	>40
T. atroviride ^{P*}	VINTEC_SCI	2016	Vintec (crop protection)	Biostimulant	-	>40
T. atroviride^{NP}	AP22TRI103	2022	Soil maize field	Germany	-	>40
T. azevedoi^{NP}	IPP0320	1992	Soil	Thailand	-	>40
T. azevedoi^{NP}	AP19TRI6	2019	Corncobs	KWS	-	>40
T. azevedoi^{NP}	AP19TRI7	2019	Corncobs	Germany	-	>40
T. brevicompactum^{NP}	AP22TRI129	2022	Soil maize field	Germany	-	>40
T. cerinum^{NP}	AP22TRI112	2022	Soil maize field	Germany	-	>40
T. gamsii ^{P*}	AP23TRI278	2023	Maize stalks	Germany	-	>40
T. gamsii ^{NP}	AP23TRI283	2023	Corncobs	Germany	-	>40
T. gamsii ^{P*}	AP23TRI285	2023	Corncobs	Germany	-	>40
T. gamsii ^{P*}	AP23TRI286	2023	Corncobs	Germany	-	>40
T. gamsii ^{NP}	AP24TRI434	2024	Soil maize field	Germany	-	21.16 ± 0.33
T. hamatum^{NP}	AP22TRI127	2022	Soil maize field	Germany	-	>40
T. harzianum^{NP}	AP19TRI12	2019	Corncobs	Germany	-	>40
T. harzianum^{NP}	AP19TRI14	2019	Maize stalks	Germany	-	>40
T. koningii^{NP}	IPP1657		Spore suspension	Trichodex	-	>40
T. koningii^{NP}	T39		*A. bisporus* fruitbodies	Serbia	-	>40
T. koningiopsis^{NP}	AP22TRI98	2022	Soil	Germany	-	>40
T. paratroviride ^{P*}	VINTEC_SC1		Biostimulant	Vintec	-	>40
T. paraviridescens^{NP}	HOHUHBot		Collection Uni. Hohenheim	-	-	>40
T. peberdyi^{NP}	AP22TRI96	2022	Soil maize field	Germany	-	>40
T. peberdyi^{NP}	AP22TRI101	2022	Soil maize field	Germany	-	>40
T. simmonsii^{NP}	BIOHEALTH_T50		Biostimulant	Biohealth GmbH	-	>40
T. velutinum^{NP}	AP22TRI95	2022	Soil maize field	Germany	-	>40
T. velutinum^{NP}	AP22TRI108	2022	Soil maize field	Germany	-	>40
T. virens^{NP}	AP22TRI141	2022	Soil maize field	Germany	-	>40
Fusarium proliferatum	IPP1663		Spore suspension	Collection Karlovsky,	-	>40
F. proliferatum	90		Symptomatic corncobs	Germany	-	>40
F. graminearum	69		Symptomatic corncobs	Germany	-	>40
F. graminearum	F. G		Symptomatic corncobs	Germany	-	>40
F. graminearum	IFA66		corncobs	Germany	-	>40
F. temperatum	22.4.		corncobs	Germany	-	>40
F. temperatum	81.1		Symptomatic corncobs	Germany	-	>40
F. temperatum	F. V		corncobs	Germany	-	>40
F. subglutinans	209.4		Symptomatic corncobs	Germany	-	>40
Penicillium paneum	AP24TRI412	2024	Symptomatic corncobs	Germany	-	>40
P. brevicompactum	AP18TRI4	2018	corncobs	Germany	-	>40
Pseudogymnoascus sp.	75		corncobs	Germany	-	>40
Pseudogymnoascus sp.	80		corncobs	Germany	-	>40
P. pannorum	64		corncobs	Germany	-	>40

Legend: ^{P}: Pathogenic; ^{P*}: Moderately pathogenic; ^{NP}: Non-pathogenic on maize as tested in the greenhouse. *Trichoderma* isolates beginning with 'AP' were recently generated by Annette Pfordt and subsequently identified by Clovis Douanla-Meli. These samples are deposited under the same nomenclature in the fungal collection of the Department of Crop Sciences, Division of Plant Diseases and Crop Protection at Georg August University of Göttingen in Germany.

Table S3. Evaluation of the repeatability and reproducibility of the real-time PCR assay. Mean Ct values and standard deviations were obtained from 10 replicates for repeatability and 3 replicates for reproducibility

	Repeatability		Reproducibility	
DNA template	Mean Ct ± SD	CV (%)	Mean Ct ± SD	CV (%)
target Plasmid DNA 100xLOD (1:10.000)	26.54 ± 0.24	0.9	26.45 ± 0.19	0.72
target Plasmid DNA 10xLOD (1:100.000)	30.53 ± 0.32	1.05	30.74 ± 0.15	0.49
T. afroharzianum CBS124620 (0.1 ng/µl)	25.76 ± 0.45	1.74	24.84 ± 0.14	0.60
T. afroharzianum 159.1 (0.1 ng/µl)	27.14 ± 0.35	1.30	27.19 ± 0.23	0.84
T. asperellum 1 ng/µl	>40	-	>40	-
T. brevicompactum 1 ng/µl	>40	-	>40	-

Table S4. Evaluation of the robustness of the real-time PCR assay. A set of templates was analysed with varying PCR reaction volume or annealing temperature. Mean Ct values and standard variations were obtained from 10 replicates

Hybridization temperature	60°C	64°C	62°C	62°C
Reaction volume	12.5 µL	12.5 µL	11.25 µL	13.75 µL
DNA template (concentration)				
target Plasmid DNA 100xLOD (1:10.000)	24.03 ± 0.19	22.26 ± 0.15	24.24 ± 0.27	24.37 ± 0.18
target Plasmid DNA 10xLOD (1:100.000)	27.36 ± 0.25	25.21 ± 0.86	27.59 ± 0.22	27.91 ± 0.23
T. afroharzianum CBS124620 (0.1 ng/µl)	26.10 ± 0.21	24.17 ± 0.08	25.73 ± 0.14	26.11 ± 0.09
T. afroharzianum 159 (0.1 ng/µl)	26.59 ± 0.26	24.47 ± 0.25	26.72 ± 0.11	27.02 ± 0.24
T. asperellum 1 ng/µl	>40	>40	>40	>40
T. brevicompactum 1 ng/µl	>40	>40	>40	>40

Table S5. Evaluation of the transferability of the real-time PCR assay. A set of templates was analysed with different qPCR equipment and commercial qPCR master mixes. Mean Ct values and standard variations were obtained from 10 replicates.

	Maxima Probe/ROX qPCR Master Mix + StepOnePlus		TaKaRa Premix Ex Taq Probe qPCR + StepOnePlus		TaqMan Universal Master Mix II (Applied Biosystems) + StepOnePlus		iTaq Universal Probes Supermix (Bio-Rad) + qTower^3 thermocycler	
DNA template	Mean Ct ± SD	CV	Mean Ct ± SD	CV	Mean Ct ± SD	CV	Mean Ct ± SD	CV
target Plasmid DNA 100xLOD (1:10.000)	24.59 ± 0.43	1.75	25.71 ± 0.23	0.89	26.50 ± 0.25	0.94	29.24 ± 0.83	2.83
target Plasmid DNA 10xLOD (1:100.000)	28.49 ± 0.62	2.18	29.96 ± 0.19	0.63	30.88 ± 0.60	1.94	33.77 ± 0.98	2.90
T. afroharzianum CBS124620 (0.1 ng/µl)	24.46 ± 0.29	1.18	25.35 ± 0.19	0.75	26.43 ± 0.02	0.08	30.46 ± 0.68	3.32
T. afroharzianum 159.1 (0.1 ng/µl)	26.15 ± 0.11	0.42	26.69 ± 0.28	1.05	28.49 ± 0.14	0.49	29.92 ± 0.55	2.76
T. asperellum 1 ng/µl	>40	-	>40	-	>40	-	>40	-
T. brevicompactum 1 ng/µl	>40	-	>40	-	>40	-	>40	-
